# Responsiveness of quality of life measures in children with peripheral vascular malformations: The OVAMA project

**DOI:** 10.1016/j.jpra.2020.11.013

**Published:** 2020-11-30

**Authors:** M.M. Lokhorst, S.E.R. Horbach, M. Waner, T.M. O, C.J.M. van der Vleuten, P.I. Spuls, C.M.A.M. van der Horst

**Affiliations:** a Department of Plastic, Reconstructive and Hand Surgery, Amsterdam University Medical Centers, University of Amsterdam, the Netherlands; b Vascular Birthmark Institute of New York, Department of Otolaryngology, Lenox Hill and Manhattan Eye, Ear, and Throat Hospitals, New York, New York, U.S.A.; c Department of Dermatology, Radboud University Medical Center, Nijmegen, the Netherlands; d Department of Dermatology, Amsterdam University Medical Centers, University of Amsterdam, Amsterdam Public Health, Infection and Immunity, the Netherlands

**Keywords:** Vascular malformations, Quality of life, Responsiveness, Core outcome set, Patient-reported outcome measure, Validity

## Abstract

**Background:**

The OVAMA (**O**utcome **M**easures for **VA**scular **MA**lformations) project determined quality of life (QoL) as a core outcome domain for evaluating treatment effect in vascular malformations. To correctly evaluate treatment effect on QoL, patient-reported outcome measures (PROMs) are needed that are responsive to changes. In children with vascular malformations, we explored if two widely used PROMs were responsive to changes: the Pediatric Quality of Life Inventory (PedsQL) and the Children's Dermatology Life Quality Index (CDLQI).

**Methods:**

In an international multicenter prospective study, conservatively and invasively treated children completed the PedsQL and CDLQI at baseline and after follow-up of 6–8 weeks. At follow-up, change in health was assessed by a global rating of change (GRC) scale. Responsiveness was assessed by testing hypotheses on expected correlation strength between change scores of the PROMs and the GRC scale, and by calculating the area under the receiver operating characteristics curve (AUC). The PROMs were considered responsive if ≥75% of the hypotheses were confirmed or if the AUC was ≥0.7.

**Results:**

Twenty-nine children were recruited in three centers in the Netherlands and United States, of which 25 completed all baseline and follow-up measurements. For both the PedsQL and CDLQI, less than 75% of the hypotheses were confirmed and the AUC was <0.7.

**Discussion:**

The results suggest that these PROMs are not sufficiently responsive for evaluating treatment effect in peripheral vascular malformations. Our study emphasizes the need for assessing responsiveness before using a PROM in evaluating treatment effect.

## Introduction

Vascular malformations are rare congenital vessel anomalies. The tangles of dilated vessels often present as a visible mass leading to a distorted appearance. Apart from the cosmetic problems, patients commonly experience pain, bleeding, functional impairment and thrombotic complications.^1^, ^2^, ^3^, ^4^, ^5^ Symptoms and complications vary depending on the type, extent and localization of the lesion.

The lesions are classified according flow velocity and the types of vessels involved. Simple types include the high-flow arteriovenous malformations, and the low-flow venous, lymphatic and capillary malformations.^3^ Lesions may additionally be of combined origin, and can occur as part of different syndromes.

The anomalies may impact different aspects of quality of life (QoL), and are associated with an overall poorer QoL when compared with the general population.^6^ Since the clinical presentation varies widely, depending on subtype, localization, size, and extension, the impact on QoL varies as well, ranging from no impact at all to a very poor QoL.^6^

Many treatments are available, such as surgical excision, sclerotherapy, embolization, laser therapy, conservative management with analgesia or compression stockings, targeted medicaments, and combinations. Treatment effect is unpredictable, as both excellent and poor results are observed. However, the patient's perspective in evaluating treatment effect is often overlooked.

Different aspects of QoL, including emotional wellbeing, mobility, social functioning and other patient-reported outcomes (PROs) were recently included in the core domain set (CDS) for vascular malformations.^7^ A CDS is a minimum set of outcome domains that should be measured when evaluating treatment outcomes in a certain health condition.^8^ The CDS results from the OVAMA (**O**utcome Measures for **VA**scular **MA**lformations) project, an international consensus project which aims at uniform outcome reporting by determining *what* and *how* to measure.^2^^,^^7^

The next step is selecting or developing appropriate outcome measurement instruments for measuring the core domains, i.e. developing a core outcome measurement set (COMS).^8^^,^^9^ QoL and other PROs are measured by patient-reported outcome measures (PROMs). To correctly evaluate treatment outcome on QoL, PROMs are needed that are able to detect changes in QoL before and after treatment, which means that the PROMs must be responsive to changes.^10^^,^^11^ It is of great clinical relevance to be familiar with the term *responsiveness,* since every measurement tool must be sufficiently responsive in order to evaluate changes over time, such as before and after treatment.

Additionally, it is crucial for clinicians that research is conducted on how their treatments affect different aspects of the patient's life. This allows for clinicians to better inform the patient, which improves shared-decision making, and ultimately enables more personalized care tailored to the specific problems of the individual. Again, for this field of research to advance, a responsive PROM is needed.

In children with vascular malformations, we explored whether two widely used QoL PROMs showed responsiveness to changes in overall health regarding the vascular malformation: the Pediatric Quality of Life Inventory (PedsQL) and the Children's Dermatology Life Quality Index (CDLQI). We investigated if these PROMs can be used for assessing treatment effect and should be considered for inclusion in the COMS for peripheral vascular malformations.

## Methods

### Patients and data collection

From October 2016 until September 2017, data were collected prospectively of children under the age of 18 with a diagnosed peripheral vascular malformation at the outpatient clinics of the Amsterdam University Medical Centre in Amsterdam, Radboud University Medical Centre in Nijmegen, and the Vascular Birthmark Institute in New York. Written informed consent was obtained from all participants.

Patient and vascular malformation characteristics were extracted from the patient files. This included gender, age at start of treatment, type of vascular malformation, size (maximal diameter of <5 cm, 5–10 cm, ≥10 cm), localization (head/neck, trunk, upper extremities, lower extremities, combined), involved tissues (skin/subcutaneous tissue, muscle, organs, bone), and previous treatments.

Patients could receive conservative treatment (watchful waiting or compression stockings), or invasive treatment (including laser therapy, sclerotherapy and surgery). Follow-up measurement was 6–8 weeks post-treatment, which is customary for evaluating treatment effect in these clinics.

### Outcome measures

#### PedsQL

The PedsQL is a generic QoL PROM for children and adolescents, containing 23 items measuring four domains of QoL: ‘physical functioning’, ‘emotional functioning’, ‘social functioning’, and ‘school functioning’.^12^ Additionally, a composite ‘psychosocial’ score can be derived. Higher scores indicate a better QoL.

#### CDLQI

The CDLQI is a PROM measuring the impact of skin disease on QoL in children. The questionnaire consists of 10 items forming 6 scales: ‘symptoms and feelings’, ‘leisure’, ‘school or holidays’, ‘personal relationships’, ‘sleep’, and ‘treatment’.^13^ Higher scores indicate a worse QoL.

All change scores were calculated such that a positive change score reflects improvement.

### Global rating of change scale

At follow-up, the children additionally filled in a global rating of change (GRC) scale for measuring experienced change since baseline in overall health regarding their vascular malformation. The question was as follows: ‘To what extent has your overall health with respect to your vascular malformation changed when compared to your situation when you filled out the previous questionnaire? In other words, has anything changed since the last time you filled out the questionnaire (concerning the vascular malformation or any complaints you might have had)?’ Response options included ‘very much worse’, ‘worse’, ‘somewhat worse’, ‘no change’, ‘somewhat better’, ‘better’, and ‘very much better’.

All questionnaires were suitable for self-report from 8–17-year old. Parents were allowed to help with completing the GRC scale.

### Evaluating responsiveness

The first method for evaluating responsiveness used the ‘construct approach’. This involves testing predefined hypotheses on correlations with other outcome measurement instruments, in this case: the other PROM and the GRC scale. For both the PedsQL and the CDLQI, we formulated 7 hypotheses (Table 1). These hypotheses were based on previous studies assessing responsiveness and methodology guidelines by COSMIN (**CO**nsensus-based **S**tandards for the Selection of Health **M**easurement **IN**struments).^11^^,^^14^, ^15^, ^16^, ^17^ As advised, if ≥75% of the hypotheses were confirmed, the questionnaire was considered responsive.^11^^,^^14^

**Table 1:** Hypotheses for testing responsiveness of the PedsQL and CDLQI total scores. If ≥75% of these hypotheses were confirmed, it was considered responsive to change.

**PedsQL**
1. High positive correlation (>0.5) between PedsQL total change score and the GRC scale
2. High positive correlation (>0.5) between PedsQL total change score and CDLQI total change score
3. Moderate positive correlation (0.3–0.5) between PedsQL total change score and the CDLQI leisure subscale change score
4. Low positive (<0.3) or negative correlation between PedsQL total change score and the CDLQI treatment subscale change score
5. Patients indicating improvement on the GRC scale should have a positive mean change score
6. Patients indicating worsening on the GRC scale should have a negative mean change score
7. The mean change score of patients indicating improvement should be higher than the mean change score of unchanged patients, which in turn should be higher than the mean change score of worsened patients
**CDLQI**
1. High positive correlation (>0.5) between CDLQI total change score and the GRC scale
2. High positive correlation (>0.5) between CDLQI total change score and PedsQL total change score
3. Moderate positive correlation (0.3–0.5) between CDLQI total change score and the PedsQL physical subscale change score
4. Low positive (<0.3) or negative correlation between CDLQI total change score and the PedsQL school subscale change score
5. Patients indicating improvement on the GRC scale should have a positive mean change score
6. Patients indicating worsening on the GRC scale should have a negative mean change score
7. The mean change score of patients indicating improvement should be higher than the mean change score of unchanged patients, which in turn should be higher than the mean change score of worsened patients

The hypotheses were formulated beforehand by two independent researchers (M.L., S.H.). Disagreement was resolved by consensus (M.L., S.H.). Spearman's rank correlation coefficients were calculated for assessing correlation strength. Correlation strength was interpreted as high (≥0.5), moderate (0.3–0.5), and low (<0.3), based on previous studies and guidelines for assessing responsiveness.^11^^,^^14^, ^15^, ^16^, ^17^, ^18^ Hypotheses 5, 6, and 7 concerned the mean change scores of improved, unchanged, and worsened patients according to the GRC scale.

With the second method, responsiveness was assessed by calculating the area under the receiver operating characteristics curve (AUC). The AUC is a value representing the instrument's ability to discriminate between improved and unchanged patients. Patients were categorized as improved or unchanged according to their GRC scale response. With an AUC of ≥0.7, an instrument can be considered responsive.^11^^,^^19^

All data were analyzed with IBM SPSS statistics 25.0.

## Results

A total of 44 children were asked to participate of which 29 were included. Twenty-five (86%) children completed both PROMs at baseline and follow-up and completed the GRC scale.

### Baseline characteristics

An overview of the included children's baseline characteristics is presented in Table 2. No significant differences were found in baseline characteristics between ex- and included children, and between children who completed follow-up and those who did not.

**Table 2:** Baseline characteristics of the 25 children who completed the questionnaires at baseline and follow-up, and the GRC scales.

Total n=25	Mean (range)	IQR (25th–75th percentile)
Age at baseline	14 (8–17)	14 (12–17)
	Frequency	Percentage
**Gender**		
Female	21	84
**Type**		
Venous	15	60
Lymphatic	4	16
Arteriovenous	2	8
Venous, capillary	2	8
Venous, lymphatic	1	4
Arteriovenous, capillary	1	4
**Localization**		
Lower extremity	10	40
Head/neck	5	20
Upper extremity	4	16
Trunk, lower extremity	2	8
Trunk, upper extremity	2	8
Trunk	1	4
Head/neck, trunk	1	4
**Size (maximal diameter)**		
<5 cm	13	52
5–10 cm	1	4
≥10 cm	11	44
**Depth/extension**		
Skin/subcutaneous tissue, muscle	11	44
Skin/subcutaneous tissue	6	24
Skin/subcutaneous tissue, muscle, bone	3	12
Skin/subcutaneous tissue, muscle, airway involvement	2	8
Skin/subcutaneous tissue, muscle, visceral organs	1	4
Muscle	1	4
Bone	1	4
**Treatment history**		
No prior treatment	11	44
Sclerotherapy	6	24
Surgery	3	12
Elastic stockings	2	8
Embolization	1	4
Laser therapy, elastic stockings	1	4
Surgery, sclerotherapy	1	4
**Treatment during study period**		
Conservative		
Expectant management	14	56
Elastic stockings	5	20
Invasive		
Sclerotherapy	5	20
Surgery	1	4

### Descriptive data

Descriptive data of the PedsQL and CDLQI at baseline and follow-up is shown in Table 3. For all domains of both PROMs scores ranged widely from no impact to large impact.

**Table 3:** Descriptive data of the PedsQL and CDLQI. Positive change scores reflect improvement.

	All patients							Conservative treatment group							Invasive treatment group						
	N	Baseline		Follow-up		Change		N	Baseline		Follow-up		Change		N	Baseline		Follow-up		Change	
		Mean	SD	Mean	SD	Mean	SD		Mean	SD	Mean	SD	Mean	SD		Mean	SD	Mean	SD	Mean	SD
**PedsQL total**	25	78.7	16.1	78.1	16.7	–0.7	9.9	19	76.8	17.1	76.0	17.1	–0.8	10.2	6	85.0	11.7	84.6	15.2	–0.4	9.8
**PedsQL physical**	25	75.3	22.2	75.3	22.0	0.1	12.0	19	73.2	23.1	73.1	22.9	0.0	11.0	6	81.8	19.1	82.3	18.7	0.5	16.2
**PedsQL emotional**	25	78.0	17.4	76.4	19.2	–1.6	19.0	19	77.9	18.4	73.2	20.6	–4.7	20.0	6	78.3	15.4	86.7	8.8	8.3	12.1
**PedsQL social**	25	85.0	16.0	85.0	17.4	0.0	11.5	19	82.1	17.0	83.9	17.5	1.8	11.2	6	94.2	7.4	88.3	18.1	–5.8	11.6
**PedsQL school**	25	78.8	18.2	77.2	19.1	–1.6	12.7	19	76.1	19.3	75.5	19.4	–0.5	12.7	6	87.5	11.3	82.5	18.9	–5.0	13.4
**PedsQL psychosocial**	25	80.6	15.2	79.5	16.1	–1.1	11.0	19	78.7	16.5	77.5	16.7	–1.1	12.0	6	86.7	8.4	85.8	13.5	–0.8	8.0
**CLDQI total**	25	6.44	5.75	6.08	6.08	0.36	3.80	19	7.32	6.12	6.47	6.17	0.84	3.59	6	3.67	3.39	4.83	6.18	–1.17	4.36
**CDLQI symptoms and feelings**	25	1.00	0.79	0.82	0.73	0.18	0.59	19	1.13	0.81	0.87	0.70	0.26	0.61	6	0.58	0.58	0.67	.88	–0.08	0.49
**CDLQI leisure**	25	0.75	0.71	0.69	0.78	0.05	0.57	19	0.75	0.68	0.67	0.72	0.09	0.55	6	0.72	0.85	0.78	1.00	–0.06	0.68
**CDLQI personal relationships**	25	0.14	0.37	0.24	0.56	–0.10	0.35	19	0.18	0.42	0.29	0.63	–0.11	0.39	6	0.00	0.00	0.08	0.20	–0.08	0.20
**CDLQI school or holidays**	25	0.92	1.04	0.80	1.04	0.12	0.67	19	1.16	1.07	0.95	1.08	0.21	0.71	6	0.17	0.41	0.33	0.82	–0.17	0.41
**CDLQI sleep**	25	0.56	0.77	0.52	0.71	0.04	0.61	19	0.74	0.81	0.58	0.77	0.16	0.60	6	0.00	0.00	0.33	0.52	–0.33	0.52
**CDLQI treatment**	25	0.44	0.77	0.56	0.92	-0.12	1.17	19	0.53	0.84	0.63	0.96	-0.11	1.24	6	0.17	0.41	0.33	0.82	-0.17	0.98

GRC scale responses are presented in Table 4. All 12 patients indicating ‘no change’ received conservative treatment. Five of 6 invasively treated patients indicated improvement.

**Table 4:** Global rating of change (GRC) scale responses.

GRC scale response (n)	–3	–2	–1	0	+1	+2	+3
All patients	0	3	3	12	2	4	1
Conservative treatment group	0	3	2	12	0	2	0
Invasive treatment group	0	0	1	0	2	2	1

No significant differences were found between different types of vascular malformation, size groups in PedsQL, CDLQI and GRC scale outcomes. The tissue involvement groups differed significantly in baseline PedsQL emotional functioning (p=0.033), school functioning (p=0.020) and baseline CDLQI symptoms and feelings (p=0.046), leisure (p<0.001) and CDLQI total score (p=0.005).

### Responsiveness: hypotheses testing and area under the receiver operating characteristics curve

Full hypotheses testing results are shown in Table 5. For both the PedsQL and CDLQI, 1 of 7 (14%) hypotheses was confirmed. The AUC for the change in total score of the PedsQL was 0.375 (95% confidence interval (CI): 0.101–0.649), and for the CDLQI 0.429 (CI: 0.155–0.702).

**Table 5:** The exact values on which the hypotheses for evaluating responsiveness were confirmed or rejected. Values in bold indicate a confirmed hypothesis. Definition of the hypotheses is shown in Table 1. GRC scale=global rating of change scale, mean change improved=the mean change score of the patients indicating improvement on the GRC scale, mean change worsened=the mean change score of the patients indicating worsening on the GRC scale, AUC=area under the receiver operating characteristics curve for discriminating between unchanged and improved patients.

	Spearman's rank correlation coefficients				5. Mean change improved (n=7)	6. Mean change unchanged (n=12)	7. Mean change worsened (n=6)	AUC	Hypotheses confirmed
	1. GRC scale	2. CDLQI total	3. CDLQI leisure	4. CDLQI treatment					
**PedsQL**	0.19	0.45	0.17	**0.25**	–1.7	1.9	–4.5	0.38 (0.10–0.65)	14%

	Spearman's rank correlation coefficients				5. Mean change improved (n=7)	6. Mean change unchanged (n=12)	7. Mean change worsened (n=6)	AUC	Hypotheses confirmed

1. GRC scale	2. PedsQL total	3. PedsQL physical	4. PedsQL school

**CLDQI**	–0.10	0.45	**0.31**	0.30	–1.00	0.67	1.33	0.43 (0.16–0.70)	14%

### Correlation between PedsQL and CDLQI score changes and GRC scale scores

Spearman's rank correlation coefficients between the PedsQL and CDLQI score changes and the GRC scale are shown in Supplementary File 1. All correlations were low, except for a moderate correlation between the CDLQI ‘personal relationships’ scale and the GRC scale.

### Correlation between PedsQL change scores and CDLQI change scores

Spearman's rank correlation coefficients between the PedsQL score changes and the CDLQI score changes are shown in Supplementary file 2. All correlations were low or moderate, except between the PedsQL ‘emotional’ scale and the CDLQI total score, which was high.

## Discussion

This exploratory study suggests that the PedsQL and CDLQI are insufficiently responsive for measuring vascular malformation-related health problems in children. Clinicians and researchers should be aware that changes in PedsQL and CDLQI scores over time might not reflect the true change these patients experienced. Responsiveness was assessed using two methods, both suggesting that these PROMs might not be suitable for evaluating treatment effects in children with vascular malformations, and thereby should not be included in the COMS.

It is notable that the group who indicated ‘no change’ on the GRC scale had a broad range of PROM change scores, suggesting the PROMs have large measurement errors (insufficient reliability), which might explain insufficient responsiveness. A study on responsiveness of two generic QoL measures in adult patients with vascular malformations also found insufficient responsiveness, plausibly caused by large measurement errors.^20^ A systematic review on outcome measurement instruments in vascular malformations found no other studies on responsiveness in this patient group or similar groups.^21^

It is known that most generic PROMs have large measurement errors, because they are often designed for cross-sectional use in very large populations.^22^ Most of the widely used generic PROMs make use of classical-test theory, for which many questions per measured domain are necessary to reduce measurement error.^23^ This is, however, limited by question burden, especially when measuring multiple health domains.

We believe researchers should be aware of this, and not blindly use a generic PROM for interpreting treatment effects without first ensuring if its responsiveness is evaluated correctly. Even the best-known PROMs are often not evaluated for responsiveness.^24^

Insufficient responsiveness could also be found if the GRC scale did not measure change correctly. However, as one would expect, the invasively treated group indicated more improvement with the GRC scale than the conservatively treated group, which predominantly indicated no change, supporting the GRC scale's ability in measuring change.

Although differences were seen between the different tissue involvement groups in baseline scores, no differences were seen between these groups in GRC scale scores. This suggests that tissue involvement might be a predictor of symptom severity and QoL, while it has no influence on treatment effects. However, we must not draw firm conclusions, since the subgroups in these analyses are very small.

Another possible cause is that treatment effect of vascular malformations is too subtle to detect, or the follow-up period was too short. However, this was contradicted by the GRC scale responses, since 71% of the patients indicating improvement rated their change as +2 or +3. Additionally, the goal is to find responsive PROMs with which all clinically relevant effects of treatment of vascular malformations can be measured.

Most correlations between PedsQL and CDLQI subscales were low to moderate, suggesting the two PROMs do not measure the same domains (convergent validity). The slightly higher correlations between the two PROMs as opposed to with the GRC scale suggest that health regarding vascular malformations might be attributed to other domains than the ones measured by the PedsQL and CDLQI. It might be that not all relevant items for this population are included (content validity), even though the domains match with the core domains for vascular malformations.

Mean scores of both PROMs at baseline show that the children's QoL was impacted moderately. However, QoL ranged broadly from normal to very poor, which may be caused by the heterogeneity of the disease, or the measurement error of the PROMs. Evidence is lacking for what clinical characteristics are associated with poor QoL. If reporting standards would be developed, it will be possible to identify clinically distinct groups, allowing the study of more homogeneous groups.

The study population seems small; however, in a recent guideline, there is explicitly no statement on sample size for evaluating responsiveness, since it involves evaluation of correlations in which significance plays no part.^15^ Additionally, our goal with this exploratory study was to investigate whether these widely used PROMs showed promising responsiveness, and therefore used the lowest cut-off values for ‘high’, ‘medium’ and ‘low’ correlations. Regarding the rarity of the disease, our goal is to find a PROM that is responsive in smaller study populations.

Innovations such as PROMIS (Patient-Reported Outcomes Measurement Information System) make use of item-response theory, reducing the number of needed items and decreasing measurement error, which is tested in different patient populations.^23^^,^^25^ We believe PROMIS might be a solution, and we are evaluating responsiveness of different PROMIS item banks in children with vascular malformations for measuring the more ‘universal’ core domains. For the remaining disease-specific domains, a disease-specific PROM is currently developed. Disease-specific domains, such as vascular malformation symptoms and appearance, might be targeted more directly by therapy, and therefore might be more suitable for detecting changes over time.

### Conclusion

This exploratory study suggests that the PedsQL and CDLQI are not sufficiently responsive for evaluating treatment effect in children with peripheral vascular malformations. Our study casts doubts on the applicability of PROMs in evaluating treatment effect in specific conditions if the measurement properties, especially responsiveness, are not evaluated. Since PROMs are increasingly used to evaluate treatment effect, it is crucial that clinicians and researchers know about the responsiveness of the PROMs they use. Otherwise, treatment effects might be wrongly assessed. Many PROMs are not developed for smaller patient populations, hence the responsiveness will be problematic in such study populations and daily care. The solution might lie in developing a PROM focusing on disease-specific health aspects, and concurrent use of innovations such as the thoroughly evaluated PROMIS for measuring more universal health aspects.

## Declaration of Competing Interest

None.

## Appendix Supplementary materials

Image, application 1

Image, application 2
