# Supplementary material for: Responsiveness of quality of life measures in children with peripheral vascular malformations: The OVAMA project
Source: JPRAS Open. 2020 Nov 30;27:70–9. doi: 10.1016/j.jpra.2020.11.013 (PMC7753079; doi:10.1016/j.jpra.2020.11.013)
Supplement: Supplementary file 1 [file mmc1.docx]

Supplementary file 1. The Spearman’s rank correlation coefficients between the PedsQL and CDLQI total score changes, and the global rating of change (GRC) scale.

|  | **Correlation with GRC scale** |
| --- | --- |
| **PedsQL total** | 0.19 |
| **PedsQL physical** | 0.15 |
| **PedsQL emotional** | 0.055 |
| **PedsQL social** | -0.21 |
| **PedsQL school** | 0.15 |
| **PedsQL psychosocial** | 0.13 |
| **CLDQI total** | -0.10 |
| **CDLQI symptoms and feelings** | -0.14 |
| **CDLQI leisure** | -0.26 |
| **CDLQI personal relationships** | 0.35 |
| **CDLQI school or holidays** | 0.030 |
| **CDLQI sleep** | -0.15 |
| **CDLQI treatment** | -0.074 |
