# Supplementary material for: Responsiveness of quality of life measures in children with peripheral vascular malformations: The OVAMA project
Source: JPRAS Open. 2020 Nov 30;27:70–9. doi: 10.1016/j.jpra.2020.11.013 (PMC7753079; doi:10.1016/j.jpra.2020.11.013)
Supplement: Supplementary file 2 [file mmc2.docx]

Supplementary file 2. The Spearman’s rank correlation coefficients between the PedsQL score changes and the CDLQI score changes.

|  | **CDLQI total** | **CDLQI symptoms and feelings** | **CDLQI leisure** | **CDLQI personal relationships** | **CDLQI school or holidays** | **CDLQI sleep** | **CDLQI treatment** |
| --- | --- | --- | --- | --- | --- | --- | --- |
| **PedsQL total** | 0.45 | 0.40 | 0.17 | 0.44 | 0.23 | 0.29 | 0.24 |
| **PedsQL physical** | 0.31 | 0.39 | 0.14 | 0.29 | 0.31 | 0.19 | 0.23 |
| **PedsQL emotional** | 0.51 | 0.17 | 0.34 | 0.31 | -0.007 | 0.33 | 0.086 |
| **PedsQL social** | 0.31 | 0.37 | 0.15 | 0.21 | -0.066 | 0.30 | 0.244 |
| **PedsQL school** | 0.30 | 0.32 | 0.11 | 0.36 | 0.31 | 0.064 | 0.27 |
| **PedsQL psychosocial** | 0.47 | 0.34 | 0.20 | 0.46 | 0.040 | 0.33 | 0.21 |
